# Association between total bilirubin and bone mineral density level in adolescents

**DOI:** 10.1186/s12891-022-05592-3

**Published:** 2022-07-04

**Authors:** Jing Wu, Jiali Su, Yangyang Wang, Jianfeng Chen, Yuanyuan Shang, Jing Li

**Affiliations:** 1 Department of Cadre Ward 2, the Second Affiliated Hospital of Harbin Medical University, Harbin, 150001 China; 2 Department of Cadre Ward 3, the Second Affiliated Hospital of Harbin Medical University, Harbin, 150001 China; 3 Department of Experimental Animal Center, the Second Affiliated Hospital of Harbin Medical University, Harbin, 150081 China; 4 Department of Neurosurgical Ward, the Second Affiliated Hospital of Harbin Medical University, Harbin, 150081 China; 5 Department of Cadre Ward 2, the Second Affiliated Hospital of Harbin Medical University, Harbin, 150081 China; 6 Department of Traditional Chinese Medicine, The Second Affiliated Hospital of Harbin Medical University, Harbin, 150081 China

**Keywords:** Total bilirubin, Bone mineral density, Adolescents, Cross-sectional

## Abstract

**Background:**

Increasing bone mass accumulation in adolescence and obtaining greater peak bone mass is one of the effective methods to prevent osteoporosis in the future. We aimed to examine the association between total bilirubin and bone mineral density (BMD) level in adolescents.

**Methods:**

We used the data from 2005–2010 and 2013–2014 cycles of National Health and Nutrition Examination Survey (NHANES). The BMD levels in the region of lumbar spine and femoral regions, including total femur, femoral neck, trochanter, and intertrochanter were measured. Univariable and multivariable linear regression model were used to assess the relationship between total bilirubin concentration and BMD.

**Results:**

A total of 3741 participants aged 12–19 years were ultimately included in the study. There were 1997 (53.38%) males and 1744 (46.62%) females. Univariate analysis results showed that age, sex, race, education, income, body mass index, dietary calcium intake, and diabetes were correlated with BMD levels. Compared with the lowest quartile of total bilirubin concentration, the highest quartile of total bilirubin concentration was positively associated with BMD levels in the regions of total femur (β = 0.036, 95% CI = 0.021 to 0.050, *P* < 0.001), femur neck (β = 0.030, 95% CI = 0.016 to 0.044, *P* < 0.001), trochanter (β = 0.033, 95% CI = 0.019 to 0.046, *P* < 0.001), intertrochanter (β = 0.040, 95% CI = 0.023 to 0.056, *P* < 0.001), and lumbar spine (β = 0.032, 95% CI = 0.018 to 0.045, *P* < 0.001). We also observe the same trend in sensitivity analysis (*P* for trend < 0.001).

**Conclusion:**

Our study demonstrated that total bilirubin concentration was positively associated with BMD levels in adolescents in United States. Total bilirubin concentration might be a protective marker against bone loss in adolescents.

## Introduction

Osteoporosis is a global public health problem that can reportedly begin from childhood or adolescence [1, 2]. Adolescence is widely accepted as a critical period of bone development [3]. During this period, the increase of bone mineral density (BMD) leads to a significant increase in bone mass in bone mass, and peak bone mass may be achieved in late adolescence [4–6]. Therefore, developing BMD in adolescence is crucial for adult bone mass accumulation and bone formation. Epidemiological evidence indicates that when peak bone mass increases by 5% in children and adolescents, the risk of osteoporotic fractures decreases by 40%, and when peak bone mass increases by 10%, the risk decreases by 50% [7, 8].

Bilirubin is a by-product of heme catabolism, which has been demonstrated to have anti-inflammatory and antioxidant properties [9–11]. Evidence from many studies indicates that oxidative stress may play a role in the pathogenesis of osteoporosis [12–14]. However, the association between bilirubin concentration and BMD and osteoporosis has not been well established, and several studies have reported conflicting results. A cross-sectional study including 918 women at post menopause reported that total bilirubin was positively associated with BMD and a decreased risk of osteoporosis [15]. Another study reported that in healthy middle-aged individuals without liver disease, serum total bilirubin can be used as a protective marker to prevent future bone loss [16]. By contrast, a genome-wide association study (GWAS) revealed that circulating bilirubin levels are not a causal risk factor for osteoporosis or fracture [17]. Furthermore, a study with a small sample size demonstrated that serum bilirubin levels were negatively associated with bone loss at the femoral neck [18].

However, data on the associations between bilirubin concentration and BMD among adolescents are scarce. Therefore, in this study, we investigated the association between total bilirubin concentration and BMD levels in the total femur, femur neck, femur neck, intertrochanter, and lumbar spine regions using data from the United States National Health and Nutrition Examination Survey (NHANES).

## Materials and methods

### Data source and study population

We used data from the 2005–2010 and 2013–2014 cycles of NHANES. NHANES, a complex nationwide survey, is conducted by the National Center for Health Statistics of the Centers for Disease Control and Prevention (CDC). The aim of this survey is to monitor the health and nutritional status among the US population. Information for demographic, socioeconomic, and health-related factors were ascertained through the interview component. More details information on survey design and methodology are available at http://www.cdc.gov/nchs/nhanes.htm. The NHANES protocol was are required to be evaluated by the National Center for Health Statistics (NCHS) Ethics Review Board.

In the present study, participants aged 12–19 years were eligible for analysis. Furthermore, we limited our analysis to participants with available data on total bilirubin concentration and BMD levels in the regions of total femur, femur neck, femur neck, intertrochanter, and lumbar spine. Finally, a total of 3741 participants were included in our study. Written informed consent was obtained from all participants.

### Laboratory analysis

Total bilirubin concentration (mg/dL) was measured on Beckman Synchron LX20 using a timed endpoint diazo method based on the NHANES Laboratory Procedures Manual. The basis of this method is that bilirubin reacts with diazo reagent in the presence of accelerator caffeine, benzoate and acetate to produce azobilirubin, and the absorbance change at 520 nm is monitored at a fixed time interval. The change of absorbance is directly proportional to the concentration of total bilirubin in the serum sample.

Dual-energy x-ray absorptiometry (DEXA) in NHANES was conducted to eligible survey participants at mobile examination center (MEC). The BMD (gm/cm^2^) measurement was performed by DEXA examinations. The BMD of lumbar spine and femoral regions, including total femur, femoral neck, trochanter, and intertrochanter.

The 2005–2010 DEXA scans were examined using Hologic QDR 4500A fan-beam, while those from 2013 to 2014 were measured by Hologic Discovery® A (Hologic Inc., Marlborough, MA) densitometers.

### Covariates

Demographic variables, including age, sex, race, education, and income were self-reported and derived from demographics questionnaires by trained interviewers using the Computer-Assisted Personal Interviewing (CAPI) system. Body mass index (BMI) was calculated as the ratio of weight to the square of the height (kg/m^2^). The dietary calcium intake (gm/day) was obtained based on the types and amounts of foods and beverages consumed in two nonconsecutive 24-h period before the interview. The presence of diabetes was determined by personal interview data (self-report).

### Statistical analysis

The characteristics of study participants were presented using the mean and standard deviations (SDs) (for continuous variable) or proportion (for categorical variable). Univariable and multivariable linear regression model were used to assess the relationship between total bilirubin concentration and BMD levels. The multivariate adjusted model was adjusted for age (continuous), sex (male or female), race (non-Hispanic white, black, Mexican American, other Hispanic, other race/ethnicity or missing), education (less than high school, high school, more than high school, or missing), income (less than $ 25,000, $ 25,000–74,999, more than $ 75,000, or missing), body mass index (underweight, normal weight, overweight, obese, or missing), calcium intake, and diabetes (yes or no). The subgroup analyses were conducted and stratified by sex. All the statistical analyses were conducted using R version 3.3.2 (http:// www.R-project.org). Two-sided *P* values < 0.05 were considered as statistical significance.

## Result

Demographic characteristics of the study population are presented in Table 1. A total of 3741 participants were ultimately included in this cross-sectional analysis. The average age of the participants was 15.46 ± 2.26 years. There were 1997 (53.38%) males and 1744 (46.62%) females. The majority of subjects were non-Hispanic white (29.24%) and Mexican American (29.56%). The average total bilirubin concentration was 0.75 ± 0.34 mg/dL.

**Table 1:** Characteristics of the participants, NHANES 2005–2010, 2013–2014 (*n* = 3741)

	Means or proportions
Age (years)	15.46 ± 2.26
Sex (%)	
Male	1997 (53.38)
Female	1744 (46.62)
Race (%)	
Non-Hispanic white	1094 (29.24)
Black	1031 (27.56)
Mexican American	1106 (29.56)
Other Hispanic	313 (8.37)
Other race/ethnicity	197 (5.27)
Education, n (%)	
Less high school	3190 (85.29)
High school	307 (8.21)
More than high school	243 (6.50)
Income (%)	
Less than $ 25,000	855 (23.54)
$ 25,000–74,999	1402 (38.60)
More than $ 75,000	1375 (37.86)
Body mass index (%)	
Underweight	493 (13.21)
Normal weight	1970 (52.77)
Overweight	709 (18.99)
Obese	561 (15.03)
Alcohol use (%)	
Never	3432 (93.39)
Past or current	243 (6.61)
Calcium intake (mg)	1000.17 ± 673.29
Total bilirubin (mg/dL)	0.75 ± 0.34
Diabetes (%)	
No	3701 (99.41)
Yes	22 (0.59)

The results of univariate analysis for risk factors of the BMD were shown in Table 2. The results showed that age, sex, race, education, income, body mass index, dietary calcium intake, and diabetes were correlated with BMD levels. Furthermore, higher total bilirubin concentration was positively associated with BMD levels in the regions of total femur, femur neck, femur neck, intertrochanter, and lumbar spine.

**Table 2:** Univariate analysis of risk factors of the bone mass density

	**Statistics**	**Total femur BMD**	**Femur neck BMD**	**Femur neck BMD**	**Intertrochanter BMD**	**Lumbar spine BMD**
		β (95% CI) *P* value	β (95% CI) *P* value	β (95% CI) *P* value	β (95% CI) *P* value	β (95% CI) *P* value
Age (year)						
< 15	1416 (37.85)	Ref	Ref	Ref	Ref	Ref
15- < 20	2325 (62.15)	0.125 (0.115, 0.135) < 0.001	0.108 (0.098, 0.117) < 0.001	0.078 (0.069, 0.086) < 0.001	0.160 (0.149, 0.171) < 0.001	0.164 (0.155, 0.173) < 0.001
Sex (%)						
Male	1997 (53.38)	Ref	Ref	Ref	Ref	Ref
Female	1744 (46.62)	-0.071 (-0.081, -0.061) < 0.001	-0.055 (-0.064, -0.045) < 0.001	-0.070 (-0.079, -0.062) < 0.001	-0.071 (-0.083, -0.059) < 0.001	0.052 (0.042, 0.062) < 0.001
Race (%)						
Non-Hispanic white	1094 (29.24)	Ref	Ref	Ref	Ref	Ref
Black	1031 (27.56)	0.070 (0.056, 0.083) < 0.001	0.067 (0.054, 0.080) < 0.001	0.048 (0.037, 0.060) < 0.001	0.083 (0.067, 0.099) < 0.001	0.050 (0.037, 0.064) < 0.001
Mexican American	1106 (29.56)	-0.013 (-0.027, -0.000) 0.047	-0.007 (-0.020, 0.005) 0.246	-0.024 (-0.035, -0.012) < 0.001	-0.012 (-0.028, 0.003) 0.115	-0.024 (-0.037, -0.011) < 0.001
Other Hispanic	313 (8.37)	-0.001 (-0.021, 0.019) 0.944	0.001 (-0.018, 0.020) 0.886	-0.006 (-0.023, 0.011) 0.496	0.004 (-0.019, 0.027) 0.744	-0.010 (-0.030, 0.010) 0.319
Other race/ethnicity	197 (5.27)	-0.016 (-0.040, 0.008) 0.192	-0.019 (-0.042, 0.004) 0.106	-0.015 (-0.036, 0.005) 0.148	-0.016 (-0.044, 0.012) 0.264	-0.010 (-0.034, 0.014) 0.402
Education, n (%)						
Less high school	3190 (85.29)	Ref	Ref	Ref	Ref	Ref
High school	307 (8.21)	0.081 (0.062, 0.099) < 0.001	0.069 (0.051, 0.087) < 0.001	0.045 (0.029, 0.061) < 0.001	0.105 (0.084, 0.127) < 0.001	0.113 (0.095, 0.132) < 0.001
More than high school	243 (6.50)	0.084 (0.064, 0.105) < 0.001	0.067 (0.047, 0.087) < 0.001	0.050 (0.032, 0.067) < 0.001	0.112 (0.088, 0.136) < 0.001	0.107 (0.087, 0.127) < 0.001
Income (%)						
Less than $ 25,000	855 (23.54)	Ref	Ref	Ref	Ref	Ref
$ 25,000–74,999	1402 (38.60)	-0.014 (-0.028, -0.001) 0.039	-0.008 (-0.022, 0.005) 0.204	-0.008 (-0.020, 0.003) 0.158	-0.022 (-0.038, -0.006) 0.008	-0.015 (-0.028, -0.001) 0.033
More than $ 75,000	1375 (37.86)	-0.010 (-0.024, 0.004) 0.153	-0.012 (-0.025, 0.001) 0.067	0.001 (-0.011, 0.013) 0.851	-0.016 (-0.032, 0.000) 0.054	-0.013 (-0.027, 0.000) 0.053
Body mass index (%)						
Under weight	1970 (52.77)	Ref	Ref	Ref	Ref	Ref
Normal weight	493 (13.21)	-0.151 (-0.165, -0.136) < 0.001	-0.139 (-0.153, -0.126) < 0.001	-0.109 (-0.122, -0.097) < 0.001	-0.181 (-0.198, -0.165) < 0.001	-0.158 (-0.172, -0.144) < 0.001
Overweight	709 (18.99)	0.059 (0.047, 0.071) < 0.001	0.063 (0.052, 0.075) < 0.001	0.039 (0.028, 0.049) < 0.001	0.069 (0.055, 0.084) < 0.001	0.051 (0.039, 0.063) < 0.001
Obese	561 (15.03)	0.116 (0.103, 0.130) < 0.001	0.129 (0.116, 0.141) < 0.001	0.082 (0.070, 0.094) < 0.001	0.128 (0.113, 0.144) < 0.001	0.103 (0.090, 0.116) < 0.001
Calcium intake (mg)						
Q1	1169 (31.81)	Ref	Ref	Ref	Ref	Ref
Q2	1120 (30.48)	-0.000 (-0.013, 0.013) 0.979	-0.003 (-0.015, 0.010) 0.664	0.003 (-0.008, 0.014) 0.624	-0.004 (-0.020, 0.011) 0.592	-0.018 (-0.031, -0.005) 0.007
Q3	1386 (37.71)	0.023 (0.011, 0.036) < 0.001	0.013 (0.001, 0.025) 0.033	0.028 (0.017, 0.038) < 0.001	0.022 (0.007, 0.036) 0.003	-0.011 (-0.023, 0.002) 0.086
Diabetes (%)						
No	3701 (99.41)	Ref	Ref	Ref	Ref	Ref
Yes	22 (0.59)	0.015 (-0.053, 0.083) 0.669	0.011 (-0.054, 0.075) 0.746	0.002 (-0.056, 0.060) 0.942	0.023 (-0.056, 0.102) 0.562	0.024 (-0.045, 0.093) 0.490
Bilirubin (mg/dL)						
Q1	454 (12.14)	Ref	Ref	Ref	Ref	Ref
Q2	1236 (33.04)	0.012 (-0.005, 0.029) 0.177	0.010 (-0.006, 0.027) 0.225	0.009 (-0.005, 0.024) 0.220	0.015 (-0.005, 0.035) 0.137	-0.003 (-0.020, 0.015) 0.772
Q3	1015 (27.13)	0.029 (0.011, 0.047) 0.001	0.019 (0.002, 0.036) 0.032	0.028 (0.013, 0.043) < 0.001	0.032 (0.012, 0.053) 0.002	0.000 (-0.017, 0.018) 0.979
Q4	1036 (27.69)	0.056 (0.038, 0.074) < 0.001	0.038 (0.021, 0.055) < 0.001	0.053 (0.038, 0.068) < 0.001	0.063 (0.043, 0.084) < 0.001	0.015 (-0.002, 0.033) 0.092

We took a multiple linear regression analysis to examine the adjusted association between total bilirubin concentration and BMD levels (Table 3). Compared with the lowest quartile of total bilirubin concentration, the highest quartile of total bilirubin concentration was positively associated with BMD levels in the regions of total femur (β = 0.036, 95% CI = 0.021 to 0.050, *P* < 0.001), femur neck (β = 0.030, 95% CI = 0.016 to 0.044, *P* < 0.001), trochanter (β = 0.033, 95% CI = 0.019 to 0.046, *P* < 0.001), intertrochanter (β = 0.040, 95% CI = 0.023 to 0.056, *P* < 0.001), and lumbar spine (β = 0.032, 95% CI = 0.018 to 0.045, *P* < 0.001). We also observe the same trend in sensitivity analysis (*P* for trend < 0.001).

**Table 3:** Multivariable adjusted associations among quartile of bilirubin and bone mass density

	**Total femur BMD**	**Femur neck BMD**	**Trochanter BMD**	**Intertrochanter BMD**	**Lumbar spine BMD**
	β (95% CI) *P* value	β (95% CI) *P* value	β (95% CI) *P* value	β (95% CI) *P* value	β (95% CI) *P* value
Bilirubin (Quintile)					
Q1	Ref	Ref	Ref	Ref	Ref
Q2	0.010 (-0.003, 0.024) 0.124	0.013 (-0.000, 0.026) 0.057	0.005 (-0.007, 0.018) 0.374	0.014 (-0.001, 0.029) 0.065	0.008 (-0.004, 0.021) 0.188
Q3	0.021 (0.007, 0.034) 0.004	0.018 (0.004, 0.032) 0.009	0.018 (0.005, 0.030) 0.006	0.023 (0.007, 0.039) 0.004	0.015 (0.002, 0.028) 0.026
Q4	0.036 (0.021, 0.050) < 0.001	0.030 (0.016, 0.044) < 0.001	0.033 (0.019, 0.046) < 0.001	0.040 (0.023, 0.056) < 0.001	0.032 (0.018, 0.045) < 0.001
*P* for trend	< 0.001	< 0.001	< 0.001	< 0.001	< 0.001

Adjust for: age (continuous), sex (male or female), race (non-Hispanic white, black, Mexican American, other Hispanic, other race/ethnicity or missing), education (less than high school, high school, more than high school, or missing), income (less than $ 25,000, $ 25,000–74,999, more than $ 75,000, or missing), body mass index (underweight, normal weight, overweight, obese, or missing), calcium intake, and diabetes (continuous)

Table 4 shows the adjusted association between total bilirubin concentration and BMD levels in different sex. Compared with the lowest quartile of total bilirubin concentration, the highest quartile of total bilirubin concentration was positively associated with BMD levels in both male and female participants, and the associations were stronger for males than females. Corresponding ORs for BMD levels among males in the regions of total femur, femur neck, femur neck, intertrochanter, and lumbar spine were 0.038 (95% CI = 0.013 to 0.063), 0.036 (95% CI = 0.012 to 0.060), 0.034 (95% CI = 0.011 to 0.057), 0.040 (95% CI = 0.012 to 0.069), 0.035 (95% CI = 0.013 to 0.058), respectively.

**Table 4:** Multivariable adjusted associations among quartile of bilirubin and bone mass density by sex

	**Total femur BMD**	**Femur neck BMD**	**Trochanter BMD**	**Intertrochanter BMD**	**Lumbar spine BMD**
	β (95% CI) *P* value	β (95% CI) *P* value	β (95% CI) *P* value	β (95% CI) *P* value	β (95% CI) *P* value
**Male**					
Bilirubin (Quintile)					
Q1	Ref	Ref	Ref	Ref	Ref
Q2	0.017 (-0.008, 0.043) 0.182	0.024 (-0.001, 0.048) 0.058	0.011 (-0.012, 0.034) 0.348	0.019 (-0.009, 0.048) 0.188	0.019 (-0.003, 0.042) 0.096
Q3	0.029 (0.004, 0.055) 0.024	0.028 (0.003, 0.052) 0.027	0.027 (0.004, 0.050) 0.023	0.030 (0.001, 0.059) 0.043	0.024 (0.001, 0.046) 0.039
Q4	0.038 (0.013, 0.063) 0.003	0.036 (0.012, 0.060) 0.004	0.034 (0.011, 0.057) 0.003	0.040 (0.012, 0.069) 0.006	0.035 (0.013, 0.058) 0.002
*P* for trend	< 0.001	0.005	< 0.001	0.001	< 0.001
**Female**					
Bilirubin (Quintile)					
Q1	Ref	Ref	Ref	Ref	Ref
Q2	0.003 (-0.011, 0.017) 0.690	0.004 (-0.010, 0.018) 0.586	-0.000 (-0.014, 0.013) 0.946	0.007 (-0.010, 0.023) 0.424	-0.002 (-0.016, 0.012) 0.814
Q3	0.008 (-0.008, 0.023) 0.348	0.009 (-0.007, 0.025) 0.278	0.004 (-0.010, 0.019) 0.548	0.010 (-0.008, 0.028) 0.29	0.003 (-0.012, 0.019) 0.683
Q4	0.021 (0.003, 0.038) 0.020	0.018 (0.000, 0.035) 0.048	0.022 (0.006, 0.038) 0.007	0.022 (0.002, 0.042) 0.032	0.015 (-0.002, 0.032) 0.091
*P* for trend	0.016	0.036	0.005	0.033	0.063
*P* for interaction	0.116	0.177	0.123	0.146	0.059

Adjust for: age (continuous), race (non-Hispanic white, black, Mexican American, other Hispanic, other race/ethnicity or missing), education (less than high school, high school, more than high school, or missing), income (less than $ 25,000, $ 25,000–74,999, more than $ 75,000, or missing), body mass index (underweight, normal weight, overweight, obese, or missing), calcium intake, and diabetes (continuous)

## Discussion

In this study, we investigated the associations between total bilirubin concentration and BMD levels among adolescents aged 12–19 years in the United States. The results revealed that total bilirubin concentration was positively associated with BMD levels in the total femur, femur neck, femur neck, intertrochanter, and lumbar spine regions. Studies have suggested total bilirubin to be a protective biomarker for diabetes, cardiovascular diseases, stroke, and peripheral arterial disease [19–22]. Increasing bone mass accumulation during adolescence and achieving higher peak bone mass can prevent future osteoporosis. Our study agrees with findings that total bilirubin may be a protective marker against bone loss in adolescents.

Our results revealed a positive correlation between bilirubin and BMD, which may be attributed to several possible mechanisms. First, oxidative stress can have detrimental effects on bone metabolism. Reactive oxygen species promote bone resorption by directly stimulating osteoclast differentiation or indirectly increasing the expression of NF-κB ligand receptor activator in osteoblasts [23, 24], and reactive oxygen species inhibit osteogenesis by regulating redox-sensitive signal pathways [25]. Total bilirubin is an effective antioxidant and can eliminate various forms of free radical oxygen species and inhibit the oxidation of lipids and lipoproteins [15, 26]. Furthermore, osteoporosis caused by oxidative stress may be related to endothelial dysfunction and decreased blood flow in bone tissue [27]; bilirubin can inhibit the activation and dysfunction of vascular endothelium in response to proinflammatory stress [28]. Second, bilirubin plays an antiinflammatory and immunomodulatory role; it protects the body from endotoxin-induced inflammation, down-regulating the expression of adhesion molecules and inhibiting the infiltration of inflammatory cells [9, 10]. In addition, proinflammatory cytokines are key mediators of inflammatory responses and have been demonstrated to regulate bone metabolism [29]. Interleukin-6 (IL-6), which is produced by osteoblasts, monocytes, and T cells, has been reported to promote osteoclast differentiation and activation [30]. Furthermore, IL-6 is related to the pathogenesis of various metabolic bone diseases [31, 32]. Tumor necrosis factor-α (TNF-α) has been reported to be involved in nontumor-induced osteopenia as well as in stimulating bone resorption and inhibiting bone formation [33, 34].

Similar to that presented in the present study, several lines of evidence have suggested that total bilirubin was positively associated with BMD in adults [15, 16]. However, conflicting results have been reported regarding the associations between total bilirubin and BMD levels. Animal experiments and population-based studies indicated that hyperbilirubinemia is not the main factor influencing BMD in patients with chronic liver disease [35]. Bagur et al. also reported no significant association between high bilirubin levels and lumbar BMD in patients with primary biliary cirrhosis [36]. Furthermore, several studies have demonstrated a significant negative correlation between total bilirubin and BMD levels [18, 37]. A cross-sectional, case–control pilot study observed that patients with Gilbert’s syndrome characterized by elevated unconjugated bilirubin tended to have decreased BMD, particularly among older individuals [38]. These inconsistent associations may have been caused by differences in study populations. Most studies with inconsistent findings have only included patients with chronic liver diseases and primary biliary cirrhosis. Because total bilirubin concentration is a key indicator of liver dysfunction, the degree of hepatocyte injury, and prognosis, the results of the aforementioned studies may have been significantly affected by the severity of liver disease in the study populations.

Notably, several studies have revealed a U-shaped association between total bilirubin concentration and coronary heart disease [39, 40], telomere length [41], and all-cause mortality [42]. These findings suggest bilirubin’s threshold effect on bone health. Furthermore, bilirubin may exert a protective effect against oxidative stress–related diseases under normal physiological conditions. When the total bilirubin level exceeds the normal physiological range, it may lead to harmful consequences, including disturbed bone formation related to osteoblast dysfunction. This also explains the significant negative correlation between increased bilirubin and BMD in people with chronic liver disease [16]. Ruiz-Gaspa et al. verified this finding and reported that high concentrations of bilirubin (5.8 mg/dL) can lower the viability, differentiation, and mineralization of osteoblasts [43].

This is the first study, to our knowledge, to demonstrate associations between total bilirubin and BMD levels among adolescents. The results have suggested a weak but significant positive association between bilirubin and BMD level. Furthermore, our analysis was based on data from a large, nationally representative survey, in contrast to those in other studies. Because raising bilirubin levels is not recommended, using a randomized controlled trial to explore the associations between total bilirubin levels and osteoporosis is impossible. Therefore, further high-quality prospective cohort studies are required to verify our findings. The present study also has several limitations. First, the cross-sectional research design precluded causal analysis. Second, although multiple covariates were adjusted for, our findings may have been affected by residual confounding confounders, such as drug therapy, physical activity, genetical factors, and potential chronic liver diseases.

In conclusion, the present study demonstrated a significant positive association between total bilirubin concentration and BMD levels in the total femur, femur neck, femur neck, intertrochanter, and lumbar spine regions among adolescents aged 12–19 years in the United States. Total bilirubin concentration may be a protective marker against bone loss in adolescents.

## Data Availability

The datasets analyzed during the current study are available in the website of the NHANES: https://www.cdc.gov/nchs/index.htm.
